# Should we monitor psychotropic drug levels in pregnancy and the postpartum period to reduce risks of recurrence?

**DOI:** 10.1192/j.eurpsy.2021.142

**Published:** 2021-08-13

**Authors:** A. Wieck

**Affiliations:** Greater Manchester Mental Health Foundation Trust, University of Manchester, Manchester, United Kingdom

**Keywords:** psychotropics, pregnancy, therapeutic drug monitoring

## Abstract

Pregnancy is associated with profound changes in pharmacokinetic processes. This is an important - and until recently neglected - area of research since the majority of women take drugs during pregnancy in addition to vitamin and dietary supplements. Recent evidence has emerged that the changes do not only include absorption, distribution and excretion but also drug metabolism, such as modifications in the regulation of hepatic metabolism and conjugation. Oestradiol is known to have an important role in the expression of cytochrome P450 isoenzymes and glucuronosyltransferase that are involved in the metabolism of psychotropic drugs. Recent studies of different psychotropic drug classes have shown that this can result in profound changes of plasma concentrations that commence early in pregnancy and gradually increase towards delivery. In this presentation pregnancy-induced metabolic changes that have been found so far for several psychotropic drugs will be discussed and the question addressed whether and how we should monitor plasma levels in our pregnant patients.

**Disclosure:**

No significant relationships.

